# Decreased m6A Modification of CD34/CD276(B7-H3) Leads to Immune Escape in Colon Cancer

**DOI:** 10.3389/fcell.2021.715674

**Published:** 2021-07-08

**Authors:** Yiran Zhou, Haodong Zhou, Jianlin Shi, Aoran Guan, Yankun Zhu, Zongliu Hou, Ruhong Li

**Affiliations:** ^1^Key Laboratory of Tumor Immunological Prevention and Treatment of Yunnan Province, First Department of General Surgery, Yan’an Affiliated Hospital of Kunming Medical University, Kunming, China; ^2^Key Laboratory of Tumor Immunological Prevention and Treatment of Yunnan Province, Department of Thoracic Surgery, Yan’an Affiliated Hospital of Kunming Medical University, Kunming, China; ^3^Key Laboratory of Tumor Immunological Prevention and Treatment of Yunnan Province, Kunming, China

**Keywords:** N6-methyladenosine, colon cancer, CD34, CD276(B7-H3), immune escape

## Abstract

Previous studies have reported that m6a modification promotes tumor immune escape by affecting tumor microenvironment (TME). Due to the complexity of TME, a single biomarker is insufficient to describe the complex biological characteristics of tumor and its microenvironment. Therefore, it is more meaningful to explore a group of effective biomarkers reflecting different characteristics of cancer to evaluate the biological characteristics of solid tumors. Here, the immune gene CD34/CD276 with different m6A peak was obtained by m6A sequencing (MeRIP-seq) of colon cancer (CRC)clinical samples and combined with MsIgDB database, which was used to perform cluster analysis on TCGA-COAD level 3 data. The CD34/CD276 as a molecular marker for CRC prognosis was confirmed by survival analysis and immunohistochemical assay. Further bioinformatics analysis was carried out to analyze the molecular mechanism of CD34/CD276 affecting the TME through m6a-dependent down-regulation and ultimately promoting immune escape of CRC.

## Introduction

CRC is the second most common cancer in men and the third most common cancer in women worldwide. Immune evasion caused by tumor progression is the most important factor leading to the death of CRC patients (Dekker et al., 2019). While immunotherapy for cancer has been shown to have significant efficacy, only a small number of patients tend to experience clinical benefits, mainly due to the tumor immunosuppressive microenvironment (Yang et al., 2019). The tumor microenvironment (TME), which consists of extracellular matrix, myofibroblasts, cytokines, fibroblasts, neuroendocrine cells, adipocytes, immune-related cells, and blood vessels (Wang et al., 2018), induces phenotypic changes of cancer cells and immune cells through complex molecular mechanisms to promote immune escape (Kaymak et al., 2021). The crosstalk between TME and immune cells is initiated and regulated by receptors on the cell surface (Clara et al., 2020). Because of the complexity of TME, a single biomarker is not enough to describe the complex biological characteristics of a tumor and its microenvironment (Rolff et al., 2016). Therefore, exploring a group of effective biomarkers reflecting the different characteristics of cancer is more meaningful for evaluating the biological characteristics of solid tumors (Hanahan and Weinberg, 2011).

N6-Methyladenosine (m6A) is considered the most abundant internal chemical modification of human mRNA. It has an important role in tumor progression by participating in RNA processing, nuclear output, translation, degradation, and RNA-protein interaction (Van Laethem et al., 2012; Wang et al., 2014; Liu et al., 2015). Many studies have reported a correlation between TME promoting tumor immune escape and m6A modification. E.g., the low expression of m6A methyltransferase WTAP is related to the high T cell-associated immune response of gastric cancer (Lin et al., 2020; Chong et al., 2021). In the mouse CRC model, the depletion of YTHDF1 strengthens the early initiation of T cells to new tumor antigens (Han et al., 2019), but the role of m6A in cancer immunity has not been extensively explored.

Herein, we obtained the immune gene CD34/CD276 with different m6A peak by m6A sequencing (MeRIP-seq) of CRC clinical samples combined with MsIgDB database. Based on TCGA database bioinformatics analysis combined with immunohistochemical verification, we confirmed that CD34/CD276 could be used as a molecular marker for CRC prognosis and promote CRC immune escape by relying on the down-regulation mechanism of m6A modification.

## Materials and Methods

### Tissue Specimen Collection

A total of 40 patients with CRC who received surgical treatment in the first department of general surgery of Yan’an Hospital Affiliated to Kunming Medical University on January 10, 2019 were selected. CRC tissues and distal paracancerous tissues were collected during the operation and stored in liquid nitrogen.

This study was approved by the Ethics Committee of our hospital. None of the patients received adjuvant chemoradiotherapy or immunotherapy before surgery, and all the tissues were pathologically confirmed as colorectal adenocarcinoma. The CRC tissues and paracancerous tissues of 10 patients were randomly selected for High-Throughput m6A Sequencing, and the CRC tissues of 40 patients were verified by immunohistochemical analysis.

### High-Throughput m6A Sequencing

Total RNA was extracted using Trizol reagent (Invitrogen, CA, United States) following the manufacturer’s procedure. The total RNA quality and quantity were analyzed using a Bioanalyzer 2100 and RNA 6000 Nano LabChip Kit (Agilent, CA, United States) with RIN number >7.0. Approximately more than 25 μg of total RNA representing a specific adipose type were used to deplete ribosomal RNA according to the instructions of Epicentre Ribo-Zero Gold Kit (Illumina, San Diego, United States). Following purification, the ribosomal-depleted RNA was fragmented into ∼100-nt-long oligonucleotides using divalent cations under elevated temperature. Then, the cleaved RNA fragments were subjected to incubated for 2 h at 4°C with m6A-specific antibody (No. 202003, Synaptic Systems, Germany) in IP buffer (50 mM Tris-HCl, 750?mM NaCl and 0.5% Igepal CA-630) supplemented with BSA (0.5 μg μl^–1^). The mixture was then incubated with protein-A beads and eluted with elution buffer (1 × IP buffer and 6.7 mM m6A). Eluted RNA was precipitated by 75% ethanol. Eluted m6A-containing fragments (IP) and untreated input control fragments were converted to the final cDNA library in accordance with a strand-specific library preparation by the dUTP method. The average insert size for the paired-end libraries was ∼100 ± 50 bp. Next, we performed the paired-end 2 × 150 bp sequencing on an Illumina Novaseq^TM^ 6000 platform at the LC-BIO Bio-tech Ltd. (Hangzhou, China) following the vendor’s recommended protocol.

### The Expression of CD276/CD34 in CRC Tissues Was Detected by Immunohistochemistry

The paraffin sections of CRC tissues were prepared, each containing two parts of the same tumor tissue, and were baked at 60°C for 120 min for dewaxing and hydration. Antigen repair was carried out under high temperature and pressure. CD274/CD34 antibody from Abcam was used, and the sections were sealed after staining. The target area of the tissue was selected with the Eclipse CI-L photo microscope for 200-fold imaging. During imaging, the tissue was tried to fill the whole field of vision to ensure the consistent background light of each photo. After imaging, Image-Pro Plus 6.0 analysis software was used to measure the cumulative optical density (IOD) of three field positives in each section with pixel area as the standard unit, as well as the corresponding tissue pixel Area; the AREAL DENSITY = IOD/Area.

### Analysis of m6A Features of Differentially Immune Genes Between CRC and Paired PCa Issues

First, Cutadapt and perl scripts in house were used to remove the reads that contained adaptor contamination, low-quality bases, and undetermined bases. Then, sequence quality was verified using Fast QC. We used HISAT2 to map reads to the genome of Homo sapiens (Version: v96) with default parameters. Mapped reads of IP and input libraries were provided for R package exomePeak, which identified m6A peaks with bed or bam format that could be adapted for visualization on the UCSC genome browser or IGV software^1^. MEME and HOME were used for *de novo* and known motif finding followed by localization of the motif with respect to peak summit by perl scripts in house. Called peaks were annotated by intersection with gene architecture using ChIPseeker. Next, StringTie was used to perform expression level for all mRNAs from input libraries by calculating FPKM {FPKM = [total_exon_fragments/mapped_reads (millions) × exon_length (kB)]}. The differentially expressed mRNAs were selected with log2 (fold change) > 1 or < −1 and *P* < 0.05 by R package edgeR.

### Target Genes Were Obtained by Matching MeRIP-Seq Results With MSIgDB Database

MSIgDB database^2^ was used to obtain immune genes match MeRIP sequencing results, and get immune genes with m6A differences peak as target genes, eventually resulting in Venn diagram.

### The Data Mining of Target Gene Is Based on the TCGA Database

Based on R 4.0.1^3^, the TCGA^4^ Level 3 RNA sequencing data and relevant clinical information of patients were downloaded. The R package “Consensus Cluster Plus” was used to Cluster all TCGA CRC patients based on the target gene. Cumulative distribution function and consistency matrix were used to determine the optimal clustering. K-M survival curves were used to compare the survival of different clusters. The gene sets of 29 kinds of immune cells were downloaded from the MSIGDB database, and an SSGSEA score was used for 29 kinds of immune cells in each sample. Ciborsort evaluated the infiltration of immune cells in different clusters, and the infiltration of different immune cells in different stages was compared. An estimate algorithm was used to calculate tumor purity in different clusters, and classical immune checkpoint molecular expression in different clusters was compared. GSEA enrichment analysis was performed on cluster samples. Finally, the target gene, GSEA optimally enriched GO, and KEGG entry gene was uploaded to the STRING database to construct a protein-protein interaction (PPI) network. A *P* < 0.05 was considered statistically significant.

## Results

### Patient Clinical Information

Among 40 patients with CRC included in the study, 18 were female with an average age of 62.39 ± 7.8 years, and 22 were male with an average age of 64.86 ± 9.44 years. There were 2 cases of TNM Stage I, 14 cases of Stage II, 12 cases of Stage II, and 12 cases of Stage IV. The detailed clinical characteristics of the subjects are shown in Table 1.

**TABLE 1:** Clinical characteristics of the studied patients.

Id	Gender	Age	Stage
Patient 1	Female	55	Stage II
Patient 2	Female	62	Stage IV
Patient 3	Male	71	Stage I
Patient 4	Female	54	Stage III
Patient 5	Male	63	Stage IV
Patient 6	Male	66	Stage II
Patient 7	Male	78	Stage II
Patient 8	Male	67	Stage II
Patient 9	Female	49	Stage III
Patient 10	Female	77	Stage IV
Patient 11	Male	56	Stage III
Patient 12	Male	79	Stage III
Patient 13	Female	67	Stage II
Patient 14	Female	73	Stage II
Patient 15	Male	71	Stage III
Patient 16	Male	73	Stage III
Patient 17	Male	69	Stage IV
Patient 18	Female	63	Stage II
Patient 19	Female	67	Stage IV
Patient 20	Female	66	Stage IV
Patient 21	Male	64	Stage II
Patient 22	Female	71	Stage II
Patient 23	Female	65	Stage III
Patient 24	Male	59	Stage I
Patient 25	Male	67	Stage IV
Patient 26	Female	54	Stage II
Patient 27	Male	70	Stage II
Patient 28	Male	55	Stage III
Patient 29	Male	59	Stage III
Patient 30	Male	76	Stage IV
Patient 31	Male	65	Stage II
Patient 32	Female	67	Stage IV
Patient 33	Female	59	Stage IV
Patient 34	Female	54	Stage III
Patient 35	Male	46	Stage II
Patient 36	Female	54	Stage IV
Patient 37	Male	43	Stage III
Patient 38	Male	72	Stage III
Patient 39	Male	58	Stage IV
Patient 40	Female	66	Stage II

### Acquire Target Gene CD34/CD276

**FIGURE 1:**
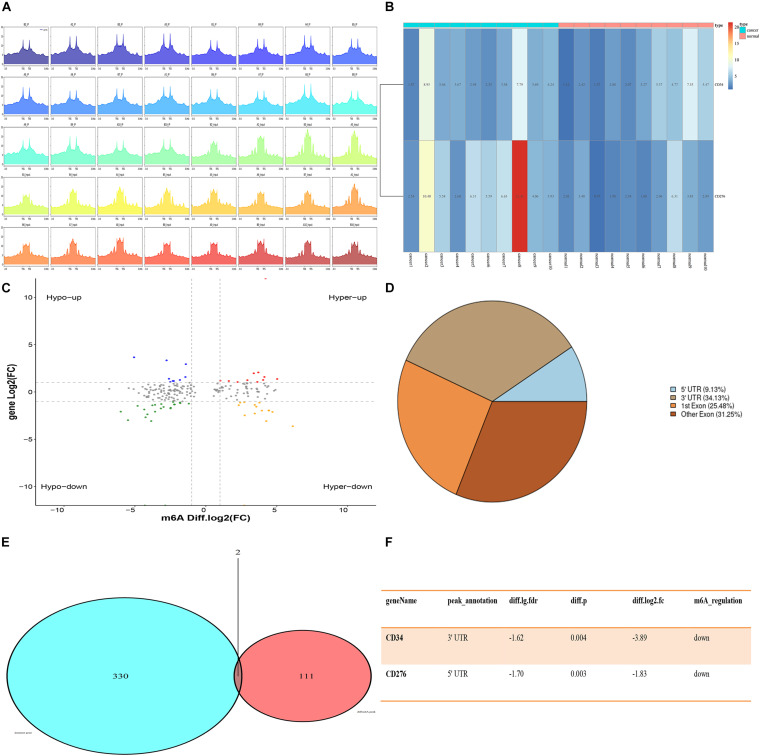
Expression of CD34/CD276 and m6A modification in CRC. **(A)** Peak statistics of clinical sequencing samples. **(B)** Compared with paracancerous tissues, the expression of CD276 was up-regulated in CRC tissue (*P* < 0.05), while the expression of CD34 did not significantly change (*P* > 0.05). **(C)** Difference m6A peak Volcano map. **(D)** Difference m6A peak calling. **(E,F)** CD34/CD276 is down-regulated m6A peak immune genes in CRC.

Quality control analysis was performed on the sequencing RAW data of CRC samples, and the quality control results were shown in Supplementary Material 1. Using the peak-calling software, the R package exomePeak performed a peak scan across the whole genome and calculated the differences between the peak groups. All regions with *P* < 0.05 were considered as a peak, and ChIPseeker was used to annotate these peaks. Finally, 111 genes with m6A differential peaks were obtained. The 330 immune-related genes were obtained from the MSIgDB database, after which the target genes were obtained by the intersection (Figure 1).

### Correlation Between Expression of CD34/CD276 and m6A Factor

**FIGURE 2:**
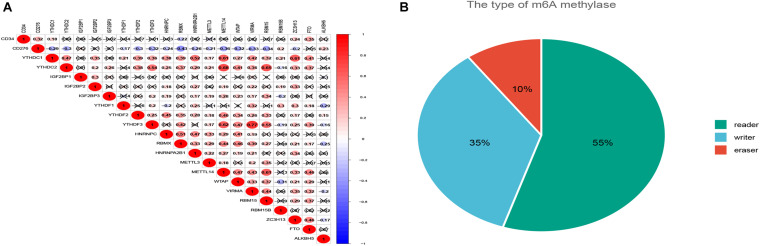
The co-expression of CD34/CD276 and m6A regulator in CRC. **(A)** The expression level of CD34 was positively correlated with the expression of FTO, while the expression level of CD276 was positively correlated with the expression of ALKBH5. **(B)** The regulatory factors of m6A were classified by function.

Twenty regulatory factors of m6A RNA methylation were collected from the literature, and the correlation between CD34/CD276 and their expression was analyzed using the TCGA database, test for association between paired genes, using one of Pearson’s product moment correlation coefficient, The results were filtered with *P* < 0.001 (Figure 2).

### Based on CD34/CD276, 472 CRC Samples in TCGA Were Clustered for Survival Analysis

**FIGURE 3:**
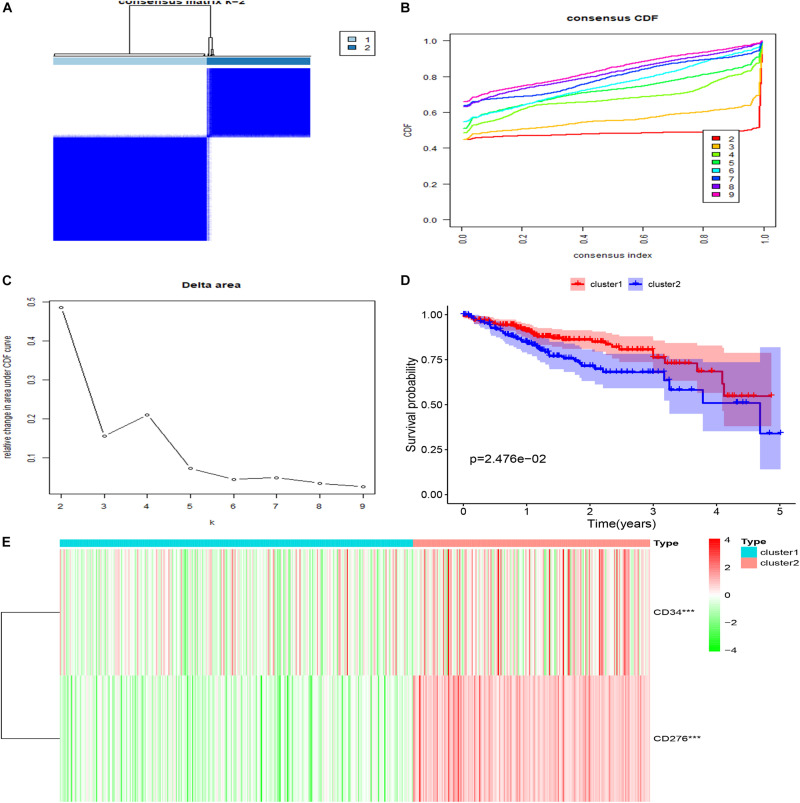
Clustering and survival analysis based on TCGA database. **(A–C)** The results of consistent cluster matrix, cumulative distribution function curve, and relative change of area under the curve showed that the expression of CD34/CD276 could be divided into two types of CRC samples. **(D)** K-M survival curve showed that patients with Cluster1 showed higher survival ability than those with Cluster2 (*P* < 0.05). **(E)** Compared with Cluster1, the expression of CD34/CD276 was up-regulated in Cluster2 (***P < 0.001).

Cluster analysis was performed on 472 patients in TCGA database using R package “ConsensusClusterPlus” according to the expression levels of CD34 and CD276, Consensus Clustering takes a subsample from a set of microarray data and determines a cluster with a specified number of clusters (k). For each k, the paired consensus values are calculated, that is, the proportion of the number of occurrences of two samples in the same subsample in the same cluster, and stored in a symmetric consensus matrix. Consensus matrices are summarized in several graphical presentations to enable users to determine a reasonable number and membership of clusters. Cluster analysis was performed on 472 patients in the TCGA database according to the expression levels of CD34 and CD276 (Consensus Cumulative Distribution Function, CDF) results show that: when *k* = 2, the consistency and clustering confidence reach the maximum, and survival analysis was performed on patients with different cluster samples. Kaplan-Meier method was used to estimate the survival time distribution of different clusters, which was presented in the form of survival curve to analyze the survival characteristics, and the survival curve between the two groups was tested by Log Rank, The results showed that the survival rate of Cluster1 group was higher than that of Cluster2 group (*P* < 0.05) (Figure 3).

### The Immune Assessment Was Conducted for Different Cluster Samples

**FIGURE 4:**
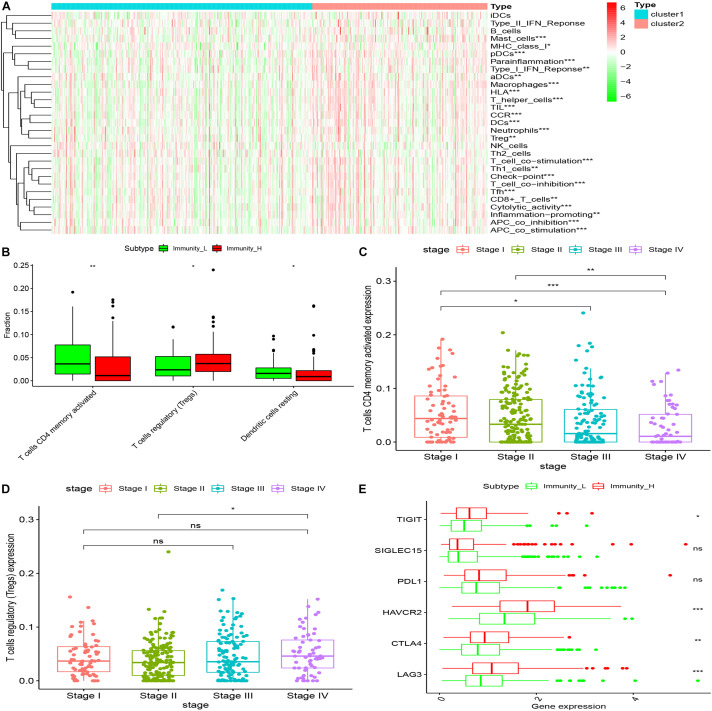
The immune components of different clusters were evaluated. **(A)** The results of SSGSEA showed that the low expression of CD34 and CD276 in the Cluster1 group was the low immune score group, while the high expression of CD34 and CD276 in the Cluster2 group was the high immune score group (*P < 0.05, **P < 0.01, ***P < 0.001). **(B)** The immune cell composition of Cluster1 and Cluster2 was further analyzed using the Ciborsort R package. The results showed that in Cluster2, the ratio of activated memory T cellsCD4 and dendritic resting cells was lower than that in Cluster1 (*P < 0.05, **P < 0.01), while the ratio of regulatory T cells was relatively higher in Cluster1 (*P < 0.05, **P < 0.01, ***P < 0.001). **(C,D)** The infiltration level of activated memory T cellsCD4 was negatively correlated with CRC TNM stage, the infiltration level of T cells (Tregs) was positively correlated with tumor TNM stage (*P < 0.05). **(E)** There was no significant difference in the expression of immune checkpoint PDL1 and SiGLEC15 between the groups with high and low immune scores (*P* > 0.05); the expression levels of TIGIT and CTLA4 were up-regulated in the group with high immune scores, and the expression levels of HAVCR2 and LAG3 were significantly up-regulated in the group with high immune scores (*P < 0.05, **P < 0.01, ***P < 0.001).

Twenty-nine immune-related genes were collected from the MSIgDB database to construct GMT files, and R package “GSVA” was used to evaluate the immunity of each tumor sample and then the enrichment scores were calculated. After normalizing the results, R Package “limma” was used for difference comparison and heat map display. The results showed that the immune score of Cluster1 group was lower than that of Cluster2 group (*P* < 0.05), Cluster1 was the group with low immune score (Immunity_L) and Cluster2 was the group with high immune score (Immunity_H) (Figure 4).

### Immune Microenvironment Assessment of Different Cluster Samples

**FIGURE 5:**
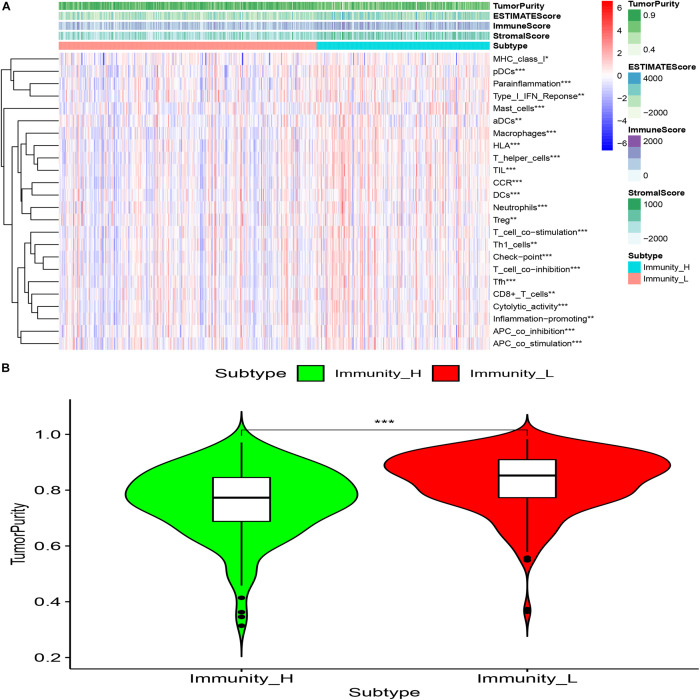
**(A)** Compared with the low immune score group, high immune array Stromal Score, Immune Score, ESTIMATE Score were higher (*p < 0.05; **p < 0.01; ***p < 0.001). **(B)** Compared with the low immune score group, the tumor purity of the high immune array was significantly reduced (***P < 0.001).

The “Estimate” R package (Estimation of Stromal and Immune cells in Malignant Tumor tissues using Expression data) was used to evaluate the tumor immune microenvironment (Gene expression score of Stromal cells) and tumor purity of different groups, The results showed that compared with the Immunity_L group, the purity of tumors in the Immunity_H group was significantly decreased (*P* < 0.001) and the content of stromal cells was increased (*P* < 0.05) (Figure 5).

### GSEA Enrichment Analysis Was Performed for Different Cluster Samples and Building of PPI Network

**FIGURE 6:**
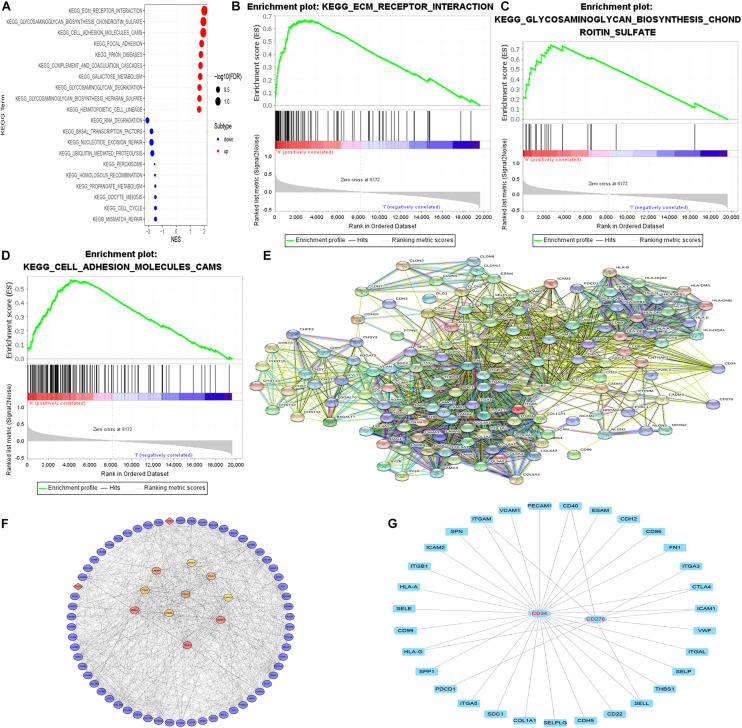
GSEA enrichment analysis and PPI network. **(A–D)** According to both FDR and NES as screening conditions, ECM receptor interaction, glycosaminoglycan biosynthesis chondroitin sulfate, and Cell adhesion molecules (CAMS) are the best KEGG enrichment items. **(E)** The string online database was used to construct a PPI network for all genes in the optimally enriched KEGG signaling pathway (high confidence = 0.7). **(F)** MCC method was used to calculate the top 10 key proteins in the PPI network. **(G)** The molecule that acts directly with CD34/CD276.

GSEA enrichment analysis was performed on tumor samples based on cluster grouping, according the normalized enrichment score (NES) and the estimated probability that the normalized enrichment score represents a false positive finding (FDR), the optimal enrichment KEGG pathway was obtained, and genes contributing to the optimally enriched KEGG signaling pathway were selected for protein-protein interaction analysis with CD34/CD276 through STRING online database, Maximal Clique Centrality (MCC) method was used to calculate the top 10 key proteins in the PPI network (using CytoHubba plug-in of Cytoscape software) (Figure 6).

### Immunohistochemistry Was Used to Evaluate the Correlation Between CD34/CD276 Expression and CRC

**FIGURE 7:**
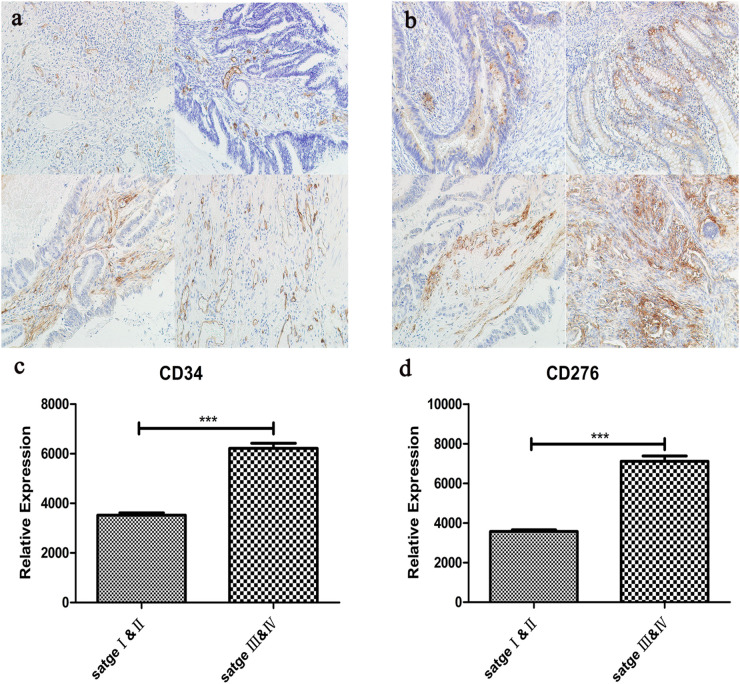
**(a)** The expression of CD34 in CRC tissues was detected by immunohistochemistry, upper left:stage I, upper right:stage II, left lower:stage III, lower right:stage IV. **(b)** The expression of CD276 in CRC tissues was detected by immunohistochemistry, upper left:stage I, upper right:stage II, left lower:stage III, lower right:stage IV. **(c)** Compared with stage I and II, CD34 expression was significantly up-regulated in stage III and IV (***P < 0.001). **(d)** Compared with stage I and II, CD276 expression was significantly up-regulated in stage III and IV (***P < 0.001).

The expression of CD34/CD276 in 40 CRC samples was measured by using the immunochemical test. The results showed that the expression of CD34/CD276 was detected in all samples, and the level of CD34/CD276 was significantly reduced in stage I and II compared with that in stage III and IV (*P* < 0.001) (Figure 7).

## Discussion

In this paper, we revealed that the immune gene CD34/CD276 could be combined as a group of molecular markers for the prognosis of CRC. We also analyzed the signal pathway and molecular mechanism of immune escape of CRC by down-regulation of m6A modification.

Many studies have reported a close relationship between the dynamic changes of M6A modification and tumor immune escape. Our results showed that in CRC, the immune gene CD34/CD276m6A modification was significantly down-regulated, the expression of CD276mRNA was up-regulated, while the expression of CD34mRNA showed no significant change. Further bioinformatics analysis based on the TCGA database revealed that cluster analysis of tumor samples based on CD34/CD276 mRNA expression could well predict the survival status of patients. CD34 antigen is a kind of cell surface glycoprotein belonging to the one-way transmembrane protein family, which is usually regarded as a marker of hematopoietic progenitor cells and has been previously used in cell therapy of various blood diseases (Hsieh et al., 2020). The evolutionary conservation of its gene sequence and protein structure in many species highlights the relevance. So far, this molecule has an important role in various cellular processes, including cell adhesion, signal transduction, and maintenance of progenitor phenotype (Inda et al., 2007; Sidney et al., 2014). However, understanding the origin of molecular development, immune regulation, and other related functions is far from complete (Díaz-Flores et al., 2020). CD276 is a new member of the B7 family of costimulatory molecules (Sun et al., 2013). It is considered as an immune costimulatory/inhibitory factor regulating the interaction between tumor and immune system (Arigami et al., 2010). The ligand of CD276 and its immunomodulatory effect have not been fully determined (Stodden et al., 2008). Although CD34/CD276 is widely expressed in mRNA in CRC, the expression of the protein is limited in steady-state, which indicates that there is an important post-transcriptional or post-translational regulatory mechanism (Kraan et al., 2014; Picarda et al., 2016; Kapoor et al., 2020). Our study confirmed that the expression of CD34 was positively correlated with FTO expression, while the expression of CD276 was positively correlated with the expression of ALKBH5. FTO/ALKBH5 was confirmed as the key “eraser” of m6A (Huang et al., 2020). The m6A modification site of CD34 is located at 3UTR, which may be related to its stability, translation, nuclear output, and cellular localization (Ke et al., 2015; Yue et al., 2018), while the m6A modification site of CD276 is located at 5UTR. It is possible that the down-regulation of m6A modification affects its ability to translate or bind to a specific RBA binding protein (RBP) (Bugaut and Balasubramanian, 2012).

The ssGSEA analysis of tumor samples with high and low expression of CD34/CD276 showed that the tumor samples with high expression of CD34/CD276 had a higher immune score, and ssGSEA was a single sample GSEA algorithm. For each sample, the immune score based on ssGSEA was obtained using immune-related gene signature and expression data. Nonetheless, the high immune score could not inhibit tumor growth, but it promoted tumor immune escape. This partly explains the lower viability of patients with high immune scores. CIBERSORT is a widely used method for high-throughput characterization of tumor-infiltrating immune cells from complex tissues (Chen et al., 2018). The temporal and spatial distribution of immune cells in the stroma around the tumor strongly affects the prognosis and treatment response. Nevertheless, this is related to the type of infiltrating immune cells because it is regulated by the unique immune editing mode of the tumor (Dunn et al., 2002). The infiltration level of regulatory T cells (Tregs) was up-regulated in the group with high expression of CD34/CD276. In TME, Tregs can be induced and differentiated by traditional T cells with a strong immunosuppressive function and promote the occurrence and development of tumors, which is the key factor of tumor immune escape (Li et al., 2020). On the other hand, the infiltration of activated memory Tcells CD4 (CD4+T cells), which promote tumor immunity, is down-regulated. CD4+T cells themselves are not the main immune subtypes of treatment, but the participation of CD4+T cells is also related to the production of effective anti-tumor response (Tay et al., 2021). Previous studies in mouse models have shown that effective response of CD8+T cells to MHCII-negative tumors requires the “helper function” of CD4+T cells. CD4+ helper T cells can inhibit the expression of inhibitory receptors in CD8+T cells and are essential for the formation of functional CD8+ memory T cells (Zhai et al., 2020). We also confirmed that the invasion of activated memory T cellsCD4 was negatively correlated with the TNM stage of CRC, while Tregs was positively correlated, which indicated that the dynamic changes of activated memory Tcells CD4 and Tregs are the key factors affecting the immune escape of CRC. In addition, we also evaluated the expression of immune checkpoints in both groups, where LAG3, HAVCR2, CTLA4, and TICIT were up-regulated in the group with high expression of CD34/CD276 (Wilky, 2019; He and Xu, 2020), which further confirmed that the high expression of CD34/CD276 drives immune escape in CRC.

The high content of unknown mixtures still limits the practicability of evaluating tumor-infiltrating immune cells. Estimate, also known as “estimating stroma and immune cells of malignant tumors using expression data,” is an algorithm for describing TME. The algorithm infers the infiltration of stromal cells and immune cells and evaluates tumor purity (Yoshihara et al., 2013). We found that the microenvironment score, matrix score, and immune score were higher, the tumor purity was significantly decreased, and the matrix content was increased in the group with high expression of CD34/CD276. This suggests that the high expression of CD34/CD276 may promote a more complex tumor extracellular microenvironment, thus promoting the immune escape of CRC, which is consistent with the best KEGG pathway for GSEA enrichment analysis. ECM receptor interaction, glycosaminoglycan biosynthesis chondroitin sulfate, and cell adhesion molecules (CAMs) signaling pathways may be the potential mechanisms of CD34/CD276 promoting immune escape of CRC. Tumor progression is characterized by dense collagen matrix accumulation and dynamic reorganization necessary to adapt to the growing invasive mass (Yamauchi et al., 2020).

In TME, T cells need to compress their nuclei in order to migrate to the sclerotic matrix, affecting gene expression, and cell mobility. In addition, nuclear compression caused by matrix stiffness leads to multiple damages to the nucleus and cell membrane during a forced passage, which eventually leads to T cell death. Intact extracellular matrix (ECM) structure and interstitial components are powerful tools for predicting the efficacy of cancer treatment (Hallmann et al., 2015; Meurette and Mehlen, 2018). ECM is a collection of interacting molecules, which is mainly composed of collagen, fibronectin, and elastin that form a matrix fiber network, and are also composed of hyaluronic acid (HA), tendon proteins, and glycoproteins containing glycosaminoglycan (GAG), thus affecting cell behavior and biomechanical and biochemical properties of the matrix (Pudełko et al., 2019; Le et al., 2020). Because cell adhesion receptors are connected to signal transduction pathways, these cell-cell interactions with ECM regulate cell phenotype, proliferation, differentiation, survival, and migration. Therefore, the changes in the expression of cell adhesion molecules and their ligands directly affect immune escape and metastasis. The abnormal expression of adhesion molecules can prevent the immune system from touching the whole tumor, a process known asendothelial anergy. This is an effective mechanism used by tumors to prevent immune cells from metastasizing to the tumor site (Darby et al., 2014; Nadanaka et al., 2018; Harjunpää et al., 2019; Läubli and Borsig, 2019; Druzhkova et al., 2020). Chondroitin sulfate (CS), as a representative sulfated glycosaminoglycan (GAG), participates in tumor immune regulation by binding to various cytokines and growth factors, cell surface receptors, adhesion molecules, enzymes, or fibrous glycoproteins in ECM, such as direct interaction with Toll-like receptors in the TME, and mediated macrophage activation. Binding to IL-8 can protect chemokines from proteolysis to stimulate angiogenesis while binding to various types of collagen can promote participation in immune cell adhesion and chemotaxis (Jalkanen and Jalkanen, 1992; Tazzyman et al., 2013; Silver and Silver, 2014; Wu et al., 2018).

We also used an online string database to construct a PPI network for genes in the optimal enrichment pathway and label 40 genes of core nodes for further exploration. While constructing the PPI network, we added CD34/CD276 gene to further evaluate its synergism. Our results showed a direct interaction between CD34/CD276 and ITGAM, CD40, IGTA3, CTLA4, ICAM1, VWF, SELL, HLA-G, and PDCD1. As an important immune checkpoint, CTLA4 has been confirmed to be significantly up-regulated in the group with high expression of CD34/CD276. This also suggests that CD34/CD276 has a synergistic effect on promoting immune escape of CRC. Finally, we further confirmed the clinical relationship between CD34/CD276 protein and CRC by IHC.

## Conclusion

In conclusion, through the clinical sample MeRIP-seq combined with MsigDB database and TGCA database data mining, we determined that CD34/CD276 can be combined as a molecular marker for predicting the viability inpatients with CRC, which may promote the immune microenvironment remodeling of CRC and ultimately promote immune escape by relying on the down-regulation of m6A modification. Although the molecular mechanism still needs to be verified by additional experiments, our findings provide a new insight into m6A modification for promoting the immune escape of CRC. Our results can provide new clues for the mechanism of CRC progression.

## Data Availability Statement

The raw data used in this study are available in the GEO database, accession no. GSE179042 (https://www.ncbi.nlm.nih.gov/geo/query/acc.cgi?acc=GSE179042, token uzahsmakzvqrxqt).

## Ethics Statement

The studies involving human participants were reviewed and approved by the Yan’an Hospital Affiliated of Kunming Medical University. The patients/participants provided their written informed consent to participate in this study.

## Author Contributions

YRZ, HZ, and JS carried out the studies, participated in collecting the data, and drafted the manuscript. AG and YKZ performed the statistical analysis and participated in its design. ZH and RL participated in the acquisition, analysis, or interpretation of the data and draft the manuscript. All authors read and approved the final manuscript.

## Conflict of Interest

The authors declare that the research was conducted in the absence of any commercial or financial relationships that could be construed as a potential conflict of interest.

## Footnotes

1 http://www.igv.org/

2 http://www.gsea-msigdb.org/gsea/index.jsp

3 https://cran.r-project.org/

4 http://cancergenome.nih.gov
