# Supplementary material for: Decreased m6A Modification of CD34/CD276(B7-H3) Leads to Immune Escape in Colon Cancer
Source: Front Cell Dev Biol. 2021 Jul 8;9:715674. doi: 10.3389/fcell.2021.715674 (PMC8297592; doi:10.3389/fcell.2021.715674)
Supplement: Supplementary file 1 [file Table_1.DOCX]

| Sample_ID | Raw_Reads | Raw_Bases | Valid_Reads | Valid_Bases | Valid% | Q20% | Q30% | GC% |
| --- | --- | --- | --- | --- | --- | --- | --- | --- |
| B2_IP | 96353902 | 14.45G | 88872782 | 11.97G | 82.85 | 96.78 | 91.82 | 47.52 |
| A2_IP | 91523118 | 13.73G | 85520642 | 11.49G | 83.72 | 96.91 | 91.97 | 47.02 |
| B3_IP | 95269336 | 14.29G | 89239792 | 11.97G | 83.75 | 97.00 | 92.09 | 48.49 |
| A3_IP | 75637236 | 11.35G | 72035866 | 9.76G | 85.99 | 97.50 | 92.74 | 48.30 |
| B1_IP | 94523420 | 14.18G | 88720016 | 11.98G | 84.47 | 96.87 | 91.89 | 47.82 |
| B4_IP | 73409710 | 11.01G | 69754532 | 9.26G | 84.10 | 97.22 | 92.30 | 49.23 |
| A4_IP | 75030314 | 11.25G | 70446844 | 9.42G | 83.69 | 97.27 | 92.37 | 49.36 |
| B5_IP | 91863912 | 13.78G | 84371628 | 11.31G | 82.10 | 97.05 | 92.24 | 48.96 |
| A5_IP | 80498876 | 12.07G | 73866548 | 9.97G | 82.58 | 97.28 | 92.41 | 48.59 |
| A6_IP | 87834998 | 13.18G | 82707924 | 11.16G | 84.72 | 97.19 | 92.22 | 47.22 |
| B7_IP | 89581078 | 13.44G | 82324158 | 11.11G | 82.66 | 97.25 | 92.33 | 47.93 |
| A1_IP | 78512374 | 11.78G | 71565460 | 9.61G | 81.60 | 97.48 | 92.75 | 47.25 |
| B6_IP | 86648002 | 13.00G | 81319338 | 10.91G | 83.97 | 97.20 | 92.27 | 47.77 |
| A7_IP | 93565740 | 14.03G | 86431630 | 11.64G | 82.95 | 97.19 | 92.26 | 48.22 |
| A8_IP | 86920308 | 13.04G | 79713960 | 10.72G | 82.21 | 97.31 | 92.45 | 48.07 |
| B8_IP | 103790542 | 15.57G | 98382772 | 13.30G | 85.42 | 97.91 | 94.13 | 47.09 |
| A9_IP | 101849724 | 15.28G | 97514326 | 13.10G | 85.76 | 98.03 | 94.34 | 47.18 |
| B9_IP | 83296166 | 12.49G | 80266076 | 10.86G | 86.89 | 98.04 | 94.39 | 48.68 |
| A10_IP | 92264894 | 13.84G | 87942574 | 11.83G | 85.50 | 98.02 | 94.36 | 47.68 |
| B10_IP | 92547822 | 13.88G | 88181228 | 11.90G | 85.71 | 98.00 | 94.31 | 47.62 |
| B2_input | 102580552 | 15.39G | 94103162 | 12.36G | 80.32 | 97.18 | 92.48 | 44.74 |
| A2_input | 82030094 | 12.30G | 76916556 | 10.12G | 82.25 | 97.51 | 92.74 | 45.09 |
| B3_input | 95283540 | 14.29G | 88254326 | 11.53G | 80.67 | 97.24 | 92.51 | 46.36 |
| A3_input | 88946840 | 13.34G | 83691868 | 10.90G | 81.69 | 97.69 | 93.10 | 45.84 |
| B1_input | 80227254 | 12.03G | 74863532 | 9.74G | 80.98 | 97.46 | 92.70 | 45.38 |
| B4_input | 82715334 | 12.41G | 77592948 | 10.09G | 81.30 | 97.52 | 92.79 | 46.30 |
| A4_input | 86517920 | 12.98G | 79834768 | 10.39G | 80.10 | 97.28 | 92.66 | 46.88 |
| B5_input | 85828136 | 12.87G | 79991820 | 10.44G | 81.12 | 97.54 | 92.84 | 46.60 |
| A5_input | 75406168 | 11.31G | 70533196 | 9.46G | 83.63 | 97.47 | 92.67 | 45.67 |
| A6_input | 98190326 | 14.73G | 91090024 | 12.15G | 82.52 | 97.19 | 92.40 | 43.95 |
| B7_input | 92341908 | 13.85G | 85396298 | 11.41G | 82.39 | 97.06 | 92.23 | 45.27 |
| A1_input | 79549562 | 11.93G | 74320256 | 9.89G | 82.91 | 97.60 | 92.91 | 45.36 |
| B6_input | 113641964 | 17.05G | 106098520 | 14.14G | 82.94 | 97.39 | 92.53 | 45.20 |
| A7_input | 90006238 | 13.50G | 84770818 | 11.25G | 83.32 | 97.45 | 92.62 | 45.59 |
| A8_input | 72167700 | 10.83G | 68003150 | 9.06G | 83.66 | 97.43 | 92.60 | 45.71 |
| B8_input | 97165626 | 14.57G | 93319802 | 12.52G | 85.88 | 98.21 | 94.58 | 43.96 |
| A9_input | 105504950 | 15.83G | 102026828 | 13.49G | 85.21 | 98.24 | 94.66 | 44.24 |
| B9_input | 101819516 | 15.27G | 97855816 | 13.03G | 85.29 | 98.26 | 94.72 | 45.65 |
| A10_input | 99891840 | 14.98G | 97178614 | 12.93G | 86.32 | 98.25 | 94.68 | 44.13 |
| B10_input | 100047992 | 15.01G | 97150062 | 12.92G | 86.09 | 98.21 | 94.62 | 44.68 |
